# Acetyl-l-Carnitine and Oxfenicine on Cardiac Pumping Mechanics in Streptozotocin-Induced Diabetes in Male Wistar Rats

**DOI:** 10.1371/journal.pone.0069977

**Published:** 2013-07-26

**Authors:** Chih-Hsien Wang, Shoei-Shen Wang, Wen-Je Ko, Yih-Sharng Chen, Chun-Yi Chang, Ru-Wen Chang, Kuo-Chu Chang

**Affiliations:** 1 Department of Surgery, National Taiwan University Hospital, Taipei, Taiwan; 2 Department of Surgery and Traumatology, National Taiwan University Hospital, Taipei, Taiwan; 3 Department of Emergency Medicine, National Taiwan University Hospital, Taipei, Taiwan; 4 Department of Physiology, College of Medicine, National Taiwan University, Taipei, Taiwan; Medical University Innsbruck, Austria

## Abstract

**Introduction:**

In the treatment of patients with diabetes, one objective is an improvement of cardiac metabolism to alleviate the left ventricular (LV) function. For this study, we compared the effects of acetyl-l-carnitine (one of the carnitine derivatives) and of oxfenicine (a carnitine palmitoyltransferase-1 inhibitor) on cardiac pumping mechanics in streptozotocin-induced diabetes in male Wistar rats, with a particular focus on the pressure-flow-volume relationship.

**Methods:**

Diabetes was induced by a single tail vein injection of 55 mg kg^−1^ streptozotocin. The diabetic animals were treated on a daily basis with either acetyl-L-carnitine (1 g L^−1^ in drinking water) or oxfenicine (150 mg kg^−1^ by oral gavage) for 8 wk. They were also compared with untreated age-matched diabetic controls. LV pressure and ascending aortic flow signals were recorded to calculate the maximal systolic elastance (*E*_max_) and the theoretical maximum flow (*Q*_max_). Physically, *E*_max_ reflects the contractility of the myocardium as an intact heart, whereas *Q*_max_ has an inverse relationship with the LV internal resistance.

**Results:**

When comparing the diabetic rats with their age-matched controls, the cardiodynamic condition was characterized by a decline in *E*_max_ associated with the unaltered *Q*_max_. Acetyl-l-carnitine (but not oxfenicine) had reduced cardiac levels of malondialdehyde in these insulin-deficient animals. However, treating with acetyl-l-carnitine or oxfenicine resulted in an increase in *E*_max_, which suggests that these 2 drugs may protect the contractile status from deteriorating in the diabetic heart. By contrast, *Q*_max_ showed a significant fall after administration of oxfenicine, but not with acetyl-L-carnitine. The decrease in *Q*_max_ corresponded to an increase in total vascular resistance when treated with oxfenicine.

**Conclusions:**

Acetyl-l-carnitine, but not oxfencine, optimizes the integrative nature of cardiac pumping mechanics by preventing the diabetes-induced deterioration in myocardial intrinsic contractility associated with unaltered LV internal resistance.

## Introduction

It has been established that diabetes results in a cardiomyopathy, and increasing evidence suggests that an altered substrate supply and utilization by cardiac myocytes could be the primary injury in the pathogenesis of this specific heart muscle disease [1], [2]. For example, patients with diabetes have an impaired cardiac glucose oxidation shifted toward a greater uptake and usage of free fatty acids (FFA) with reduced metabolic efficiency [3]. These alterations in cardiac metabolism may be responsible for both the increased susceptibility of the diabetic heart to myocardial ischemia and a proportionally greater decrease of myocardial performance [4], [5]. Thus, in the treatment of patients with diabetes, one objective is an improvement of cardiac carbohydrate metabolism to alleviate myocardial ischemia and left ventricular (LV) dysfunction [6]. The major strategies of the treatment are either reducing the circulating levels of FFA through carnitine supplementation or inhibiting the mitochondrial uptake of FFA through suppression of carnitine palmitoyltransferase-1 (CPT-1).

CPT-1, located in the outer mitochondrial membrane, is a key enzyme in FFA oxidation, and is the rate-limiting step involved in the transfer of fatty acyl groups into the mitochondria [7]. Carnitine is the essential cofactor of CPT-1, acting as the acceptor of fatty acyl groups to transport long-chain fatty acids across mitochondrial membranes for β-oxidation [8], [9]. Carnitine also reduces the intramitochondrial ratio of acetyl-CoA to free CoA, which stimulates the activity of the pyruvate dehydrogenase complex to facilitate glucose oxidation. An alternative approach to achieve a switch in energy substrate preference, away from FFA metabolism and toward glucose metabolism, is to inhibit FFA uptake by the mitochondria using CPT-1 inhibitors.

Carnitine derivatives are potent antiradical agents and may protect tissues from oxidative damage [10], [11]. Acetyl-L-carnitine (ALC) (a carnitine derivative) possesses similar physiological functions but better bioavailability and antioxidant capacity compared with carnitine [12]. The more effective action of ALC compared with l-carnitine on oxidative stress may be attributed to the acetyl group [13]. ALC was reported to have protective action on NADPH-induced lipid peroxidation of rat cardiac microsomes [14]. Moreover, long-term treatment with ALC may be of potential value in preventing the progressive loss of myocardial sympathetic nervous function in patients with diabetes [15]. Conversely, oxfenicine (OXF) is a well-characterized CPT-1 inhibitor that can reduce the accumulation of long-chain acyl-carnitine to enhance glucose metabolism [16]. Our team demonstrated in the past that ALC, but not OXF, attenuated arterial stiffening by reducing aorta levels of malondialdehyde (MDA) in insulin-deficient rats [17]. MDA is a highly toxic byproduct formed by lipid oxidation-derived free radicals, which can react with collagen to form MDA-collagen cross-links with profound cardiovascular risk [18], [19]. Thus, the crucial question yet to be answered is whether the impaired cardiac function in diabetes can be improved by OXF therapy associated with high MDA content in the diabetic heart.

The myocardium of the left ventricle is a viscoelastic material whose mechanical properties are reflected in the behavior of the ventricular chamber (i.e. the relationships among chamber pressure, volume, and flow) [20], [21]. For this study, we compared the effects of ALC and of OXF on cardiac pumping mechanics in streptozotocin (STZ)-induced diabetes in male Wistar rats, with a particular focus on the pressure-flow-volume relationship. LV pressure and ascending aortic flow signals were measured to evaluate the systolic mechanical behavior of the ventricular pump, by making use of the elastance-resistance model [22], [23]. Cardiac levels of MDA were also detected in the diabetic rats after administration of ALC or OXF.

## Methods

### Animals and Catheterization

Two-month-old male Wistar rats were randomly divided into 6 groups: (i) normal controls (NC) (*n* = 16); (ii) NC+ALC (*n* = 16); (iii) NC+OXF (*n* = 16); (iv) STZ-induced diabetic rats (DM) (*n* = 16); (v) DM+ALC (*n* = 16); and (vi) DM+OXF (*n* = 16). Diabetes was induced in animals by a single tail vein injection with 55 mg kg^−1^ STZ in 0.1M citrate buffer (pH 4.5) (Sigma Chemical Co., St. Louis, MO, USA). Blood glucose levels were determined using SURESTEP Test Strips (Lifescan Inc., Milpitas, CA, USA) for confirming developments of hyperglycemia. Two wk later, rats with stable hyperglycemia were daily treated with either ALC (Sigma Chemical Co., St Louis, MO, USA) or OXF (Sigma Chemical Co., St Louis, MO, USA). It has been suggested that treatment with l-carnitine (1 g L^−1^ in drinking water) could exert cardio-protective effects in STZ-induced diabetic rats [24]. In this study, the insulin-deficient animals were administered ALC at a dose of 1 g L^−1^, which was added to the drinking water for the duration of the study. Considering that the NC had higher body weight and lower drinking amount than the DM, we treated the NC with ALC at a dose of 3 g L^−1^ in drinking water. Two to 3 animals were housed per cage in a 12-h light/dark cycled animal room with free access to Purina chow and water. We measured the water amount the animals daily consumed per cage and calculated the water consumption per rat in average. At the end of the experiment, the DM drank 48.2±0.5 mL d^−1^, and the NC drank 23.1±0.4 mL d^−1^. In average, the dosage of ALC per rat was ∼148 mg kg^−1^ in the DM and ∼146 mg kg^−1^ in the NC. Conversely, OXF was dissolved in carboxymethylcellulose sodium salt (Sigma Chemical Co., St Louis, MO, USA) because of its poor water solubility. OXF was then delivered to rats by gavage at the doses of 150 mg kg^−1^ d^−1^. Rats were studied 8 wk after exposure to ALC or OXF to determine the drug’s effects on their systolic mechanical behavior of their ventricular pump. All animal experiments were approved by National Taiwan University’s Animal Care and Use Committee and conducted according to the *Guide for the Care and Use of Laboratory Animals*.

General surgical procedures and measurements of cardiodynamic variables in anesthetized rats have been previously described [25]. Animals were anesthetized with intraperitoneal sodium pentobarbital (50 mg kg^−1^), placed on a heating pad, intubated, and ventilated with a rodent respirator (model 131, New England Medical Instruments, Medway, MA, USA). The chest was opened through the second intercostal space of the right side. An electromagnetic flow probe (model 100 series, internal circumference 8 mm; Carolina Medical Electronics, King, NC, USA) was positioned around the ascending aorta to record the pulsatile aortic flow. A high-fidelity pressure catheter (model SPC 320, size 2F; Millar Instruments, Houston, TX, USA) was inserted via the isolated right carotid artery into the LV to measure LV pressure. The electrocardiogram (ECG) of lead II was recorded with an ECG/Biotach amplifier (Gould, Cleveland, OH, USA). The selective LV pressure and aortic flow signals averaged 5–10 beats in the time domain using the peak R wave of ECG as a fiducial point. A single-beat estimation technique was performed to calculate the systolic elastance and resistance, which characterize the pumping mechanics of diabetic hearts [26], [27].

### Prediction of the LV Pressure Using the Elastance-resistance Model

**Figure 1:**
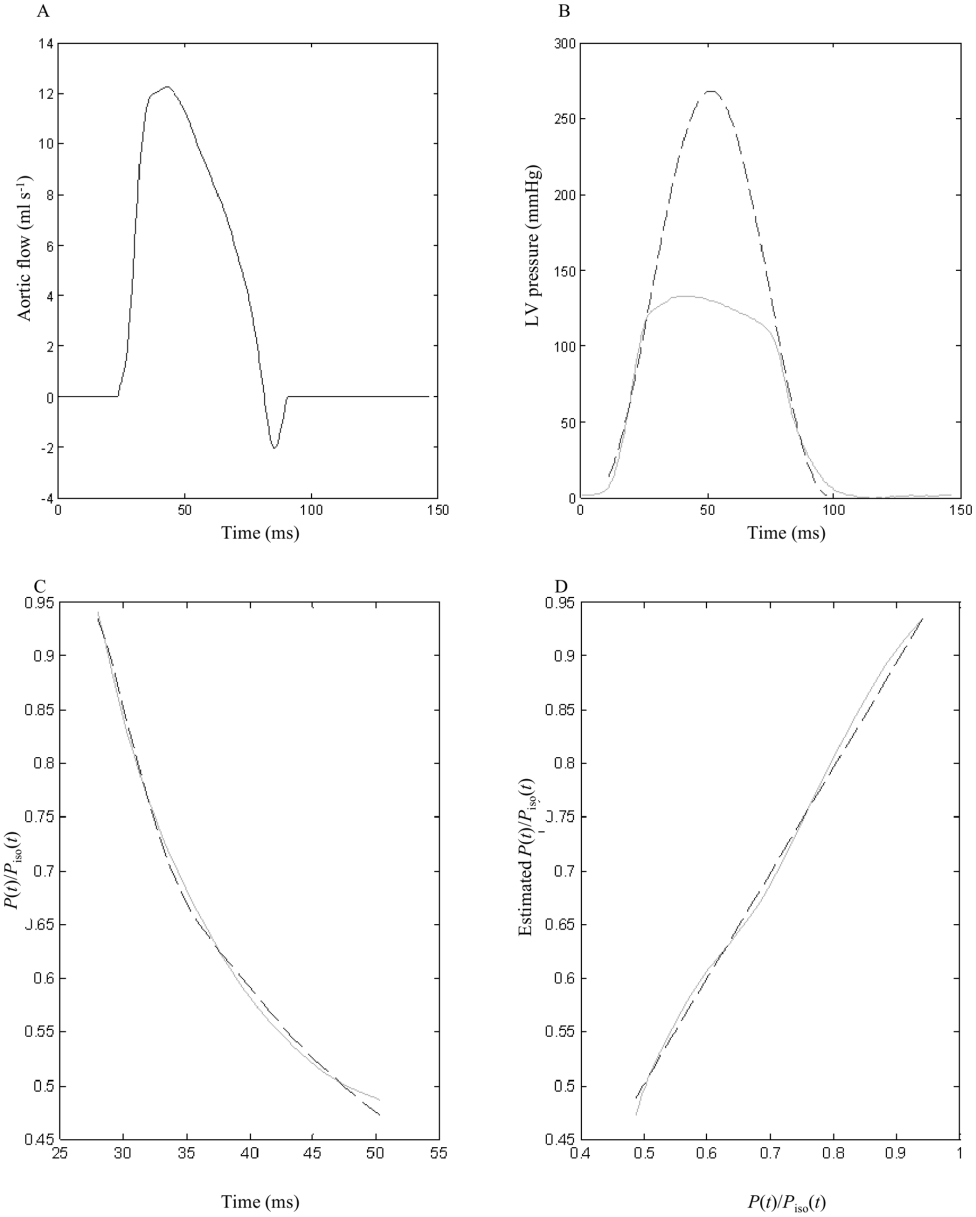
The solid lines of A and B show the measured ascending aortic flow signal and the LV pressure waveform, respectively, of one control rat. In Graph B, the dashed line represents the isovolumic pressure curve at an end-diastolic volume, which is estimated by fitting a sinusoidal function to the isovolumic portions of the measured LV pressure. Graphs C and D show the measured data and model-generated data when the elastance-resistance model is fit over *t_ej_*<*t*<*t_piso_*_max_; *t_ej_* is the onset of ventricular ejection and *t_piso_*_max_ is the time of peak isovolumic pressure. In Graph C, the solid line represents measured data, and dashed lines represent model-derived data. In Graph D, the dashed line has the slope of regression that equals 1.0. The solid line represents the relation between the measured and model-generated data. *P*(*t*) is the measured LV pressure; *P_iso_*(*t*) is the estimated isovolumic pressure; *P*(*t*)/*P_iso_*(*t*) is the ratio of *P*(*t*) to *P_iso_*(*t*).

Model-derived pressure of the LV 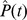 can be predicted using the elastance-resistance model if the model parameters are previously identified [22], [23]. The relationship among instantaneous LV pressure, isovolumic pressure, and aortic flow can be written as follows:
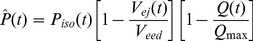
(1)where 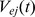 is the instantaneously ejected volume computed by numerically calculating the running integral of the aortic flow signal 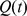. *Q*_max_ is the theoretical maximum flow, and *V_eed_* is the effective LV end-diastolic volume, which is the volume difference between LV end-diastolic volume and the zero-pressure volume-axis intercept. 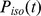 is the isovolumic pressure obtained by occluding the ascending aorta near the sinuses of the Valsalva at the end of the diastole. In this study, 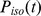 was derived from the measured pressure of an ejection contraction by using a nonlinear least-squares approximation technique [28]:
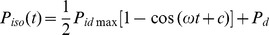
(2)where 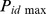 is a peak-developed isovolumic pressure, ω is an angular frequency, c is a phase shift angle of the sinusoidal curve, and 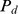 is the LV end-diastolic pressure. 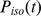 in Figure 1B is obtained by fitting the measured LV pressure curve segments from the end-diastolic pressure point to the peak 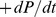 and from the pressure point of the peak 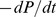 to the same level as the end-diastolic pressure of the preceding beat [29]. The peak of the ECG R wave is used to identify the LV end-diastolic point. The estimated peak isovolumic pressure 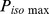 is the pressure sum of 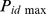 and 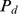.

Both *V_eed_* and *Q*_max_ are the model parameters that remain to be determined by curve-fitting techniques. Campbell *et al*. [22] found that Equation 1 can be used to fit the measured LV pressure of an ejecting beat effectively, if the fitting interval is 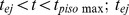 is the onset of ventricular ejection and *t_piso_*_ max_ is the time of peak isovolumic pressure. Initial values of *V_eed_* and *Q*_max_ are chosen first. Thereafter, the Nelder-Meade simplex algorithm is used to adjust *V_eed_* and *Q*_max_ iteratively to minimize the root-mean-square error (*e_p_*) [30]. Parameters coinciding with the minimum objective function are recorded as the model estimates of the systolic pumping mechanics of the LV (Figure 1C). Thus, the LV systolic elastance can be calculated by using *E*(*t*) = *P_iso_*(*t*)/*V_eed_*. Its maximal value is the maximal systolic elastance (*E*_max_ = *P_iso_*_max_/*V_eed_*). The internal resistance of the LV can be expressed as 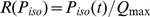 [23]. In addition, the total vascular resistance of the systemic circulation (*R_p_*) was calculated as the mean aortic pressure/mean aortic flow.

### LV End-systolic Equilibrium Point

The LV end-systolic equilibrium point could be identified as follows. The peak LV isovolumic pressure at the end-diastolic volume (*P_iso_*_max_) was estimated by the equation (2). The pressure-ejected volume loop was obtained by the time integration of aortic flow and the measured LV pressure. Thus, drawing a tangential line from the estimated *P_iso_*_max_ to the right corner of the pressure-ejected volume loop yielded a point referred to as the end-systolic equilibrium point [27], [31].

### Cardiac dP/dt_max_, dP/dt_min_, and Time Constant of LV Isovolumic Pressure Decline

Readings of pulsatile LV pressure waveform yielded cardiac *dP*/*dt*_max_, *dP*/*dt*_min_, and time constant of LV isovolumic pressure decline (τ). The LV end-diastolic point was identified as the peak of the ECG R wave. The time constant of LV pressure decay during the isovolumic relaxation period was calculated using the method proposed by Weiss et al. [32]; τ was calculated as the negative inverse slope of the ln*P* versus *t* relationship. Since the LV isovolumic pressure decline was assumed to be monoexponential, we examined the linearity of the ln*P* versus *t* relation and calculated LV τ only when the relation between ln*P* and *t* yielded a high linear correlation coefficient [33].

### Estimate of MDA Content in the LV by the use of Thiobarbituric Acid (TBA) Assay

Although MDA is not the only physiological molecule that can react with TBA [34], the TBA assay is still the most frequently used assay for MDA. Based on this method, results are “TBA reactive substances” (TBARS) instead of MDA. Hence, TBARS is used as an estimate of MDA herein.

At the end of catheterization, the rat heart was perfused with phosphate buffered saline (PBS). Thereafter, the LV was dissected, washed quickly with ice-cold PBS, and immediately frozen with liquid nitrogen. The frozen tissues were stored at −80°C until analysis. All tissues were homogenized in the RIPA buffer (Sigma Chemical Co., St Louis, MO, USA) with a 1% protease inhibitor cocktail (Sigma Chemical Co., St Louis, MO, USA) and centrifuged at 1600 *g* at 4°C for 10 min to obtain supernatants for MDA measurement. LV MDA contents were estimated by TBARS using a commercial kit (Cayman, U.S.A.) [35]. Protein concentrations of the LV were assayed using the Bradford method (DCProtein Assay, Bio-Rad) [36].

### Statistics

Results are expressed as means ± SE. Two-way ANOVA was used to assess the cardiodynamic and metabolic effects of ALC and of OXF in the STZ-induced diabetic rats. Simple effect analysis was implemented when a significant interaction between diabetes and ALC or OXF occurred. Differences among means within levels of a factor were determined using Tukey’s honestly significant difference (HSD) method. Statistical significance is defined at *p*<0.05.

## Results

Table 1 shows the effects of either ALC or OXF on blood glucose level, body weight (BW), and left ventricular weight (LVW) in the DM. The high glucose level in the DM did not change in response to either ALC or OXF treatment. After exposure to ALC, the DM showed a significant increase in BW, but did not differ in LVW from the untreated diabetic controls. The diabetes-related increase in ratio of the LVW to BW was attenuated by administration of ALC to the DM. By contrast, OXF produced no significant changes in BW, LVW, and LVW/BW in the DM. Neither ALC nor OXF therapy exerted effects on those basic variables in the NC.

**Table 1:** Effects of ALC and of OXF on blood glucose level, body weight, and left ventricular weight in the STZ-diabetic rats.

Variable	NC	NC+ALC	NC+OXF	DM	DM+ALC	DM+OXF
BS	98.8±1.5	105.8±2.9	104.6±2.0	468.3±16.8†	462.0±8.0	458.8±9.8
BW	451.9±9.1	477.5±10.9	471.3±8.4	292.1±8.1†	326.6±10.1‡	309.7±11.7
LVW	0.839±0.025	0.829±0.023	0.916±0.020	0.689±0.027†	0.635±0.022	0.746±0.024
LVW/BW	1.852±0.030	1.736±0.024	1.950±0.041	2.359±0.060†	1.944±0.031‡	2.409±0.068

All values are expressed as means ± SE. BS, blood sugar (mg dL^−1^); BW, body weight (g); LVW, left ventricular weight (g); LVW/BW, ratio of the LVW to BW (mg g^−1^). NC, normal controls; NC+ALC, NC treated with ALC; NC+OXF, NC treated with OXF; DM, STZ-diabetic rats; DM+ALC, DM treated with ALC; DM+OXF, DM treated with OXF; ALC, acetyl-L-carnitine; OXF, oxfenicine.

† Statistical difference (*P*<0.05) from the NC.

‡ Statistical difference (*P*<0.05) from the DM.

Table 2 shows the effects of either ALC or OXF on basic hemodynamic parameters in the DM. Neither ALC nor OXF prevented the diabetes-related decline in basal hear**t** rate (*HR*). By contrast, OXF but not ALC had a significant rise in mean aortic pressure and a fall in cardiac output in the DM. However, the diabetes-induced increase in cardiac index was not affected in response to treatment of the DM with either of the two drugs. Neither ALC nor OXF therapy produced effects on those hemodynamic variables in the NC.

**Table 2:** Effects of either ALC or OXF on hemodynamic parameters in the STZ-diabetic rats.

	NC	NC+ALC	NC+OXF	DM	DM+ALC	DM+OXF
*HR*	407.8±9.9	406.3±8.3	398.0±7.6	337.5±3.8†	354.1±7.4	331.9±8.2
*CO*	1.92±0.09	1.95±0.06	2.01±0.09	1.92±0.07	1.89±0.11	1.70±0.05‡
*CI*	2.04±0.10	1.97±0.06	2.09±0.08	2.63±0.07†	2.47±0.12	2.27±0.08
*MAP*	100.5±1.9	98.4±2.5	105.2±1.8	95.1±3.2	88.8±3.1	108.9±3.1‡

All values are expressed as means ± SE. *HR*, basal heart rate (beats min^−1^); *CO*, cardiac output (mL s^−1^); *CI*, cardiac index (L min^−1^ m^−2^); *MAP*, mean aortic pressure (mmHg). NC, normal controls; NC+ALC, NC treated with ALC; NC+OXF, NC treated with OXF; DM, STZ-diabetic rats; DM+ALC, DM treated with ALC; DM+OXF, DM treated with OXF; ALC, acetyl-L-carnitine; OXF, oxfenicine.

† Statistical difference (*P*<0.05) from the NC.

‡ Statistical difference (*P*<0.05) from the DM.

Table 3 shows the effects of either ALC or OXF on cardiac function in the DM. Treatment of the DM with OXF, but not ALC, produced a significant increase in LV end-systolic pressure (*P_es_*). However, neither ALC nor OXF prevented the diabetes-related augmentation in LV end-diastolic pressure (*P_ed_*). The diminished *dP*/*dt*_max_ in the DM was improved in response to either ALC or OXF treatment. The diabetes-related decline in *dP*/*dt*_min_ was attenuated by administration of ALC to the DM. ALC therapy also shortened the diabetes-derived prolongation in time constant of the LV isovolumic decay (τ). However, OXF treatment produced no beneficial effects on either *dP*/*dt*_min_ or τ in the DM. Neither ALC nor OXF therapy exerted effects on those cardiac variables in the NC.

**Table 3:** Effects of either ALC or OXF on cardiac function in the STZ-diabetic rats.

	NC	NC+ALC	NC+OXF	DM	DM+ALC	DM+OXF
*P_es_*	105.8±3.3	102.9±5.3	105.7±2.8	96.2±1.6	91.9±3.2	112.5±3.0‡
*P_ed_*	2.49±0.30	3.29±0.39	2.58±0.31	9.77±1.18†	6.19±0.70	7.52±1.08
*dP*/*dt*_max_	10420.2±462.1	10213.1±535.6	10207.1±443.5	6918.1±209.7†	8615.7±353.2‡	8220.9±317.9‡
*dP*/*dt*_min_	−7261.9±299.5	−6940.9±287.2	−6990.6±190.2	−5008.5±198.5†	−5926.9±225.4‡	−4936.6±193.5
τ	8.54±0.21	9.38±0.26	9.35±0.24	14.81±0.65†	11.13±0.38‡	14.46±0.55

All values are expressed as means ± SE. *P_es_*, LV end-systolic pressure (mmHg); *P_ed_*, LV end-diastolic pressure (mmHg); *dP*/*dt*_max_ (mmHg s^−1^); *dP*/*dt*_min_ (mmHg s^−1^); τ, time constant of LV isovolumic pressure decay (ms); LV, left ventricle. NC, normal controls; NC+ALC, NC treated with ALC; NC+OXF, NC treated with OXF; DM, STZ-diabetic rats; DM+ALC, DM treated with ALC; DM+OXF, DM treated with OXF; ALC, acetyl-L-carnitine; OXF, oxfenicine.

† Statistical difference (*P*<0.05) from the NC.

‡ Statistical difference (*P*<0.05) from the DM.

The solid curves of Figure 1A and 1B show the measured ascending aortic flow signal and the LV pressure waveform, respectively, of one control rat. In Figure 1B, the dashed line represents the isovolumic pressure curve at the end-diastolic volume, which was estimated by fitting a sinusoidal function to the isovolumic portions of the measured LV pressure. Figures 1C and 1D demonstrate the similarity between the computed and measured LV pressure waveforms during the fitting interval *t_ej_*<*t*<*t_piso_*_max_. The averaged value over all animals studied (*n* = 96) for *e_p_* as an indication of the quality of fit was 0.0041±0.0002. Goodness of model fit was also reflected in a high coefficient of determination (0.9901±0.0005) and a relatively low standard error of the estimate (2.41±0.09%). Using the elastance-resistance model, these data indicate that model parameters *V_eed_* and *Q*_max_ were of good quality for analyzing the cardiac pumping mechanics.

**Figure 2:**
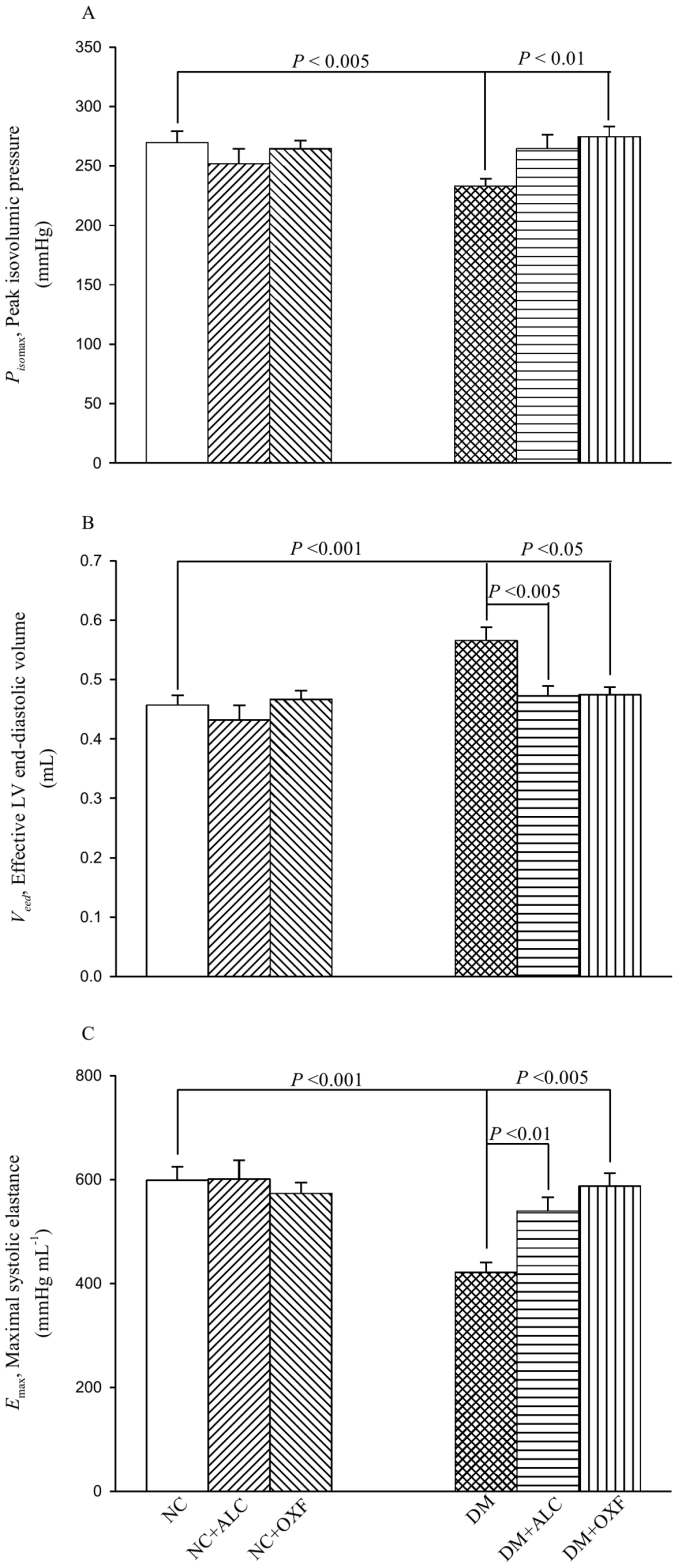
Effects of either ALC or OXF on *P_iso_*_max_ (A), *V_eed_* (B), and *E*_max_ (C) in DM. *E*_max_ can be determined by the ratio of *P_iso_*_max_ to *V_eed_*. NC, normal controls; NC+ALC, NC treated with ALC; NC+OXF, NC treated with OXF; DM, STZ-induced diabetic rats; DM+ALC, DM treated with ALC; DM+OXF, DM treated with OXF; ALC, acetyl-L-carnitine; OXF, oxfenicine; *P_iso_*_max_, the estimated peak isovolumic pressure; *V_eed_*, the effective LV end-diastolic volume; *E*_max_, the maximal systolic elastance.

Figure 2 shows the effects of ALC and of OXF on the diabetic heart’s estimated *P_iso_*_max_ (A), *V_eed_* (B), and *E*_max_ (C). In the DM group, *P_iso_*_max_ decreased compared with the NC, whereby the inversely associated *V_eed_* increased, causing a significant fall in *E*_max_. After treatment with OXF, the diabetic rats showed a significant rise in *P_iso_*_max_, a decline in *V_eed_*, and exhibited a marked increase in *E*_max_. Without affecting *P_iso_*_max_, ALC therapy also produced a benefit for *E*_max_ in the DM because of its ability to significantly diminish *V_eed_*. Neither ALC nor OXF exerted effects on those cardiodynamic variables in the NC.

**Figure 3:**
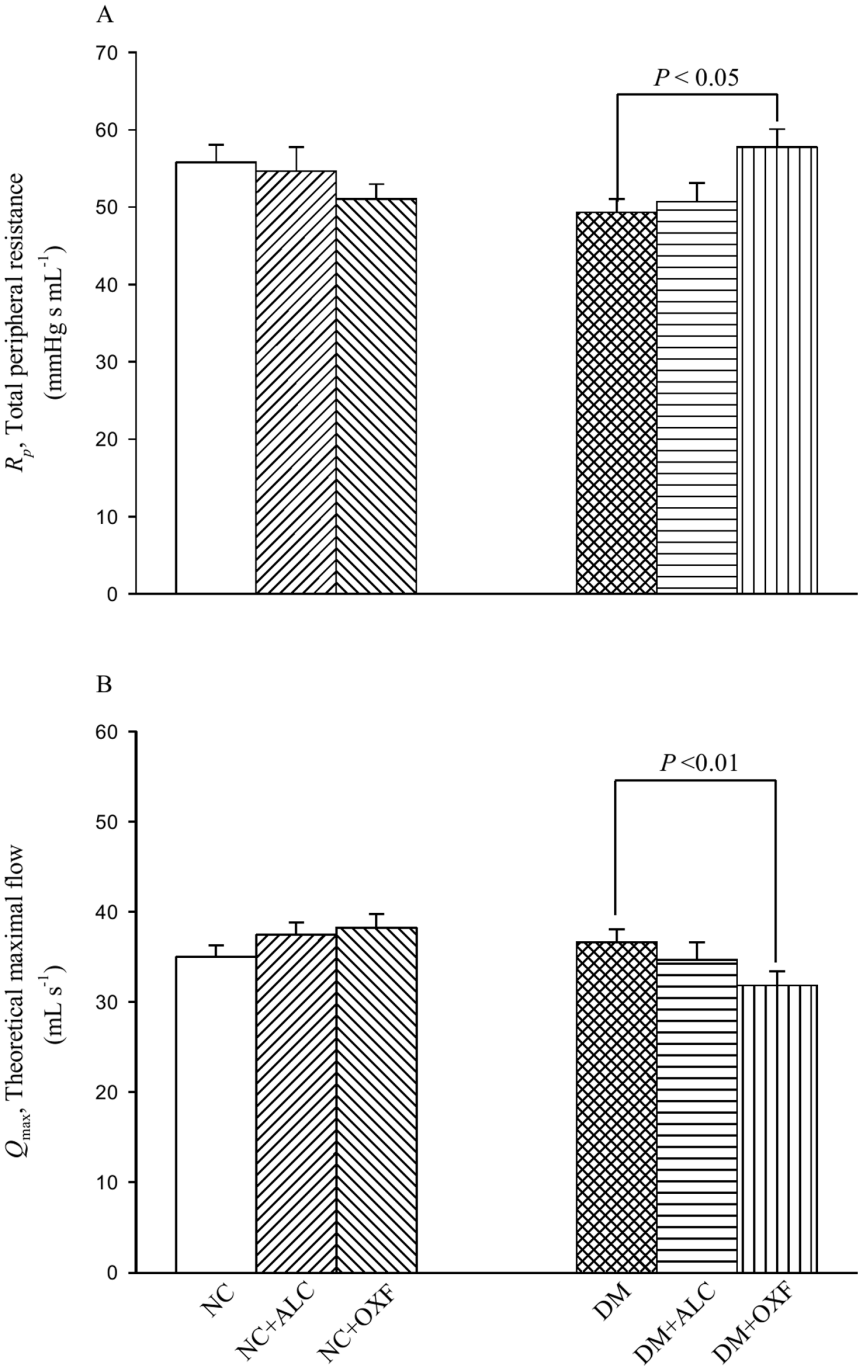
Effects of either ALC or OXF on *R_p_* (A) and *Q*_max_ (B) in DM. NC, normal controls; NC+ALC, NC treated with ALC; NC+OXF, NC treated with OXF; DM, STZ-induced diabetic rats; DM+ALC, DM treated with ALC; DM+OXF, DM treated with OXF; ALC, acetyl-L-carnitine; OXF, oxfenicine; *Q*_max_, the theoretical maximum flow; *R_p_*, the total peripheral resistance.

Figure 3 shows the effects of ALC and of OXF on *R_p_* (A) and *Q*_max_ (B) in the diabetic rats. Compared with the NC, the DM had no significant changes in both *R_p_* and *Q*_max_. After exposure to OXF, the diabetic rats showed a significant rise in *R_p_* and a fall in *Q*_max_. By contrast, ALC therapy had no impact on *R_p_* and *Q*_max_ in the DM. Neither ALC nor OXF exerted effects on *R_p_* and *Q*_max_ in the NC.

**Figure 4:**
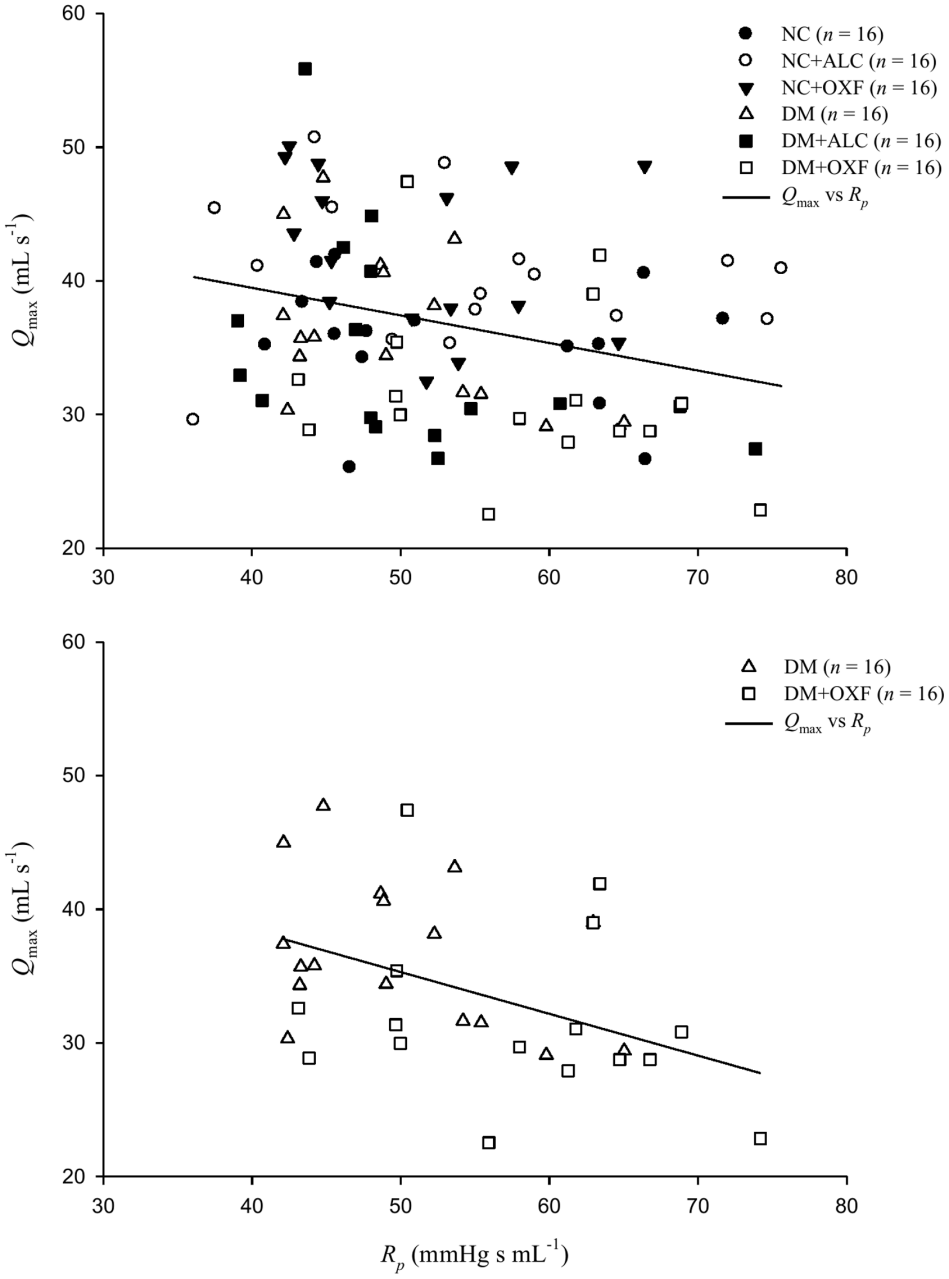
In A, the inverse relation between *Q*_max_ and *R_p_* is noted after pooling the data of all the groups (*Q*_max_ = 47.7300−0.2064× *R_p_* and *r* = 0.2909; *p*<0.005). However, the significant inverse linear correlation between the two parameters is predominantly derived from the data of the DM and DM+OXF groups (*Q*_max_ = 50.9368−0.3126× *R_p_* and *r* = 0.4390; *p*<0.05 in Figure 4B). NC, normal controls; NC+ALC, NC treated with ALC; NC+OXF, NC treated with OXF; DM, STZ-induced diabetic rats; DM+ALC, DM treated with ALC; DM+OXF, DM treated with OXF; ALC, acetyl-L-carnitine; OXF, oxfenicine; *Q*_max_, the theoretical maximum flow; *R_p_*, the total peripheral resistance.

Figure 4 shows an inverse relation between *Q*_max_ and *R_p_*, which was evident after pooling the data of all the groups (*Q*_max_ = 47.7300−0.2064× *R_p_* and *r* = 0.2909; *p*<0.005 in Figure 4A). Intriguingly, the significant inverse linear correlation between the two parameters was predominantly derived from the data of the DM and DM+OXF groups (*Q*_max_ = 50.9368−0.3126× *R_p_* and *r* = 0.4390; *p*<0.05 in Figure 4B).

**Figure 5:**
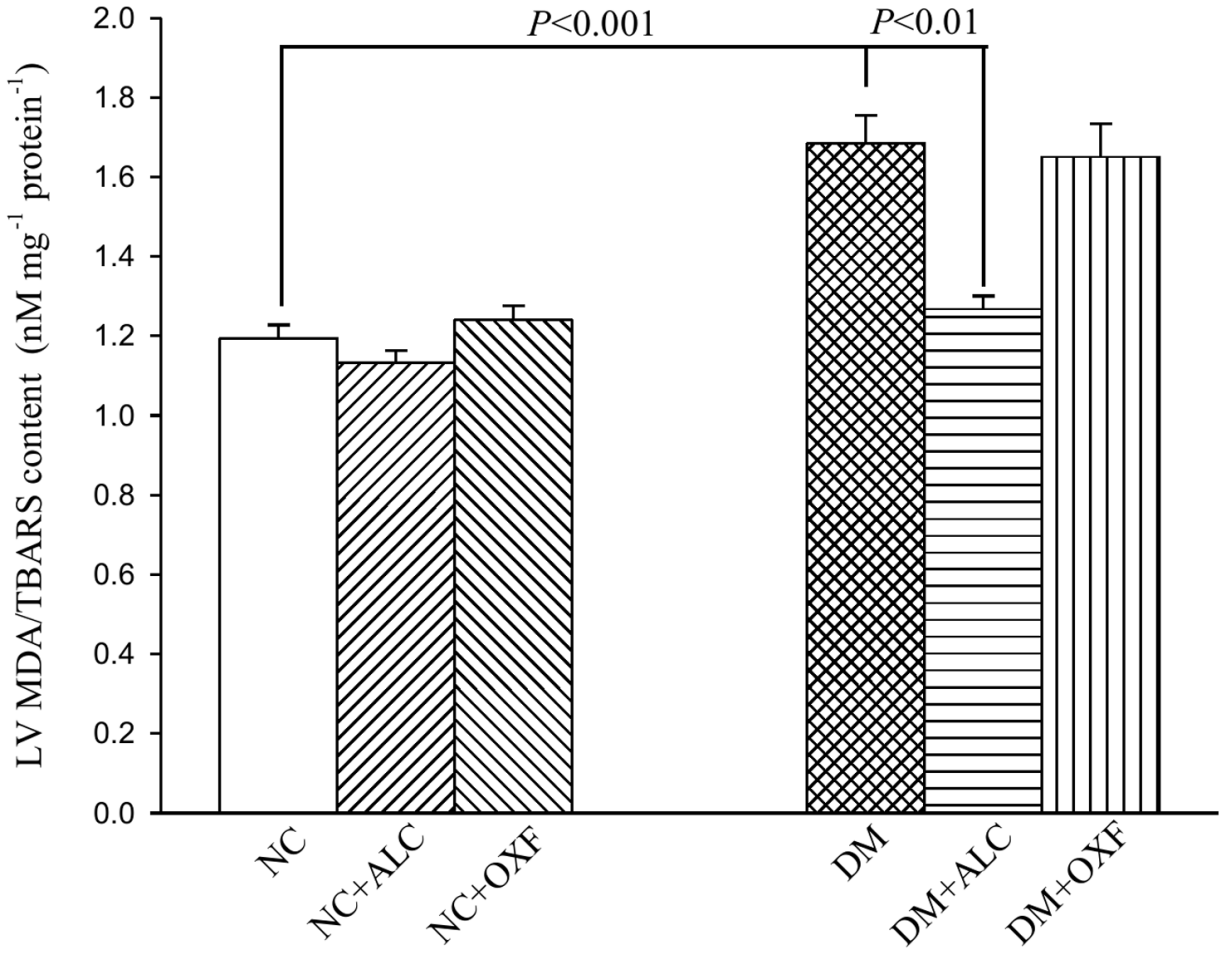
Effects of either ALC or OXF on cardiac levels of MDA/TBARS in DM. NC, normal controls; NC+ALC, NC treated with ALC; NC+OXF, NC treated with OXF; DM, STZ-induced diabetic rats; DM+ALC, DM treated with ALC; DM+OXF, DM treated with OXF; ALC, acetyl-L-carnitine; OXF, oxfenicine.

When comparing the diabetic rats with their age-matched controls, the metabolic condition was characterized by an increase in the plasma levels of FFA (shown in Ref. 17) and the cardiac levels of MDA/TBARS (Figure 5). The diabetes-related increase in plasma levels of FFA and cardiac levels of MDA/TBARS were attenuated by the administration of ALC to the diabetic rats. By contrast, treatment of the DM with OXF enhanced their already high FFA plasma levels (shown in Ref. 17). Moreover, OXF therapy produced no beneficial effects on lipid oxidation-derived MDA/TBARS of the diabetic rat heart. Neither ALC nor OXF therapy exerted effects on those metabolic factors in the NC.

## Discussion

Previous work from our laboratory demonstrated that OXF, but not ALC, elevates *R_p_* in the diabetic peripheral arteries, which parallels its increase in plasma levels of FFA [17]. By contrast, ALC attenuates the diabetes-related arterial stiffening and cardiac hypertrophy through its ability to reduce aorta levels of MDA/TABRS in the DM.

In this study, we compared the effects of ALC and of OXF on cardiac pumping mechanics in insulin-deficient rats. The systolic mechanical behavior of the ventricular pump could be characterized by both *E*_max_ and *Q*_max_ [22], [23]_._ Physically, *E*_max_ is an indicator of elasticity, which reflects subtle changes in contractile status and is independent of preload, afterload, and heart rate in a given contractile state of the ventricle [20], [37]. Therefore, *E*_max_ represents the contractility of the myocardium as an intact heart. However, *Q*_max_ has an inverse relationship with LV internal resistance and is the amount of outflow generated by the ventricle if it were to eject under zero load condition [23]. Results from this study suggest that either ALC or OXF increases *E*_max_ to protect the contractile status from deteriorating in the diabetic heart. However, OXF diminishes *Q*_max_ so that the LV internal resistance rose, impairing the ventricular outflow in the DM. The novelty of this study is that one can distinguish the effect of *Q*_max_ from that of *E*_max_ on the pumping function of the diabetic heart administered either ALC or OXF.

Abnormalities of insulin regulation in the diabetic heart may cause disturbances in calcium homeostasis and the myosin isoenzyme profile [38]–[40], which is responsible for the defects of cardiac pumping mechanics. As mentioned, LV *E*_max_ can be determined by the ratio of *P_iso_*_max_ to *V_eed_*. In this study, a significant decrease in *P_iso_*_max_ implied that the diabetic myocardium was incapable of producing enough pressure to support *E*_max_ along with the increased *V_eed_*. The worsened *P_iso_*_max_–*V_eed_* relation in the diabetic heart suggested that the underlying cooperative mechanisms in the cardiac muscle, such as length-sensitivity [41], may be impaired. Meanwhile, the shift of the myosin isoenzyme profile from the fast V_1_ isoform toward the slow V_3_ isoform has been noticed in the diabetic heart [38]–[40]. Although Shroff *et al*. [23] reported that *Q*_max_ has an inverse relationship with the percentage of slow V_3_ isoform, no significant alteration in *Q*_max_ was observed in the DM in this study.

In experimental animals, it has been shown that carnitine levels are depressed in the diabetic cardiomyopathic heart [42]. In this study, we found that treatment with ALC significantly affected the STZ-derived impairment in *V_eed_*, leading to an increase in *E*_max_. Neely and Morgan [43] suggested that higher plasma levels of FFA and their fatty acyl-CoA esters may be detrimental to the myocardial structure and function. Folden *et al*. [44] also demonstrated a novel role of MDA in lipid peroxidation and oxidative stress-associated cardiac dysfunction. Thus, the reduced plasma FFA and cardiac MDA levels from ALC may be responsible for the prevention of diabetes-related damage in the myocardial contractility. By contrast, in the absence of any significant changes in cardiac MDA content, the already high plasma levels of FFA augmented when the DM was treated with OXF [17]. OXF might accordingly be expected to exert no benefit to the diabetic heart and even worsen the contractile status of the LV. However, we found that treating the diabetic rats with OXF showed a decrease in *V_eed_* with an accompanied increase in *P_iso_*_max_, which significantly increased *E*_max_. Zarain-Herzberg and Rupp [45] reported that CPT-1 inhibitor has effects on LV function, which can be attributed to selective changes in the dysregulated gene expression of cardiomyocytes whereby the structure of several proteins are modified. Thus, its ability to increase the sarcoplasmic reticulum (SR) Ca^2+^-ATPase-2 protein expression and the SR Ca^2+^-ATPase activity may allow OXF to improve the contractile state of the diabetic heart.

Another aspect of cardiac mechanics is *Q*_max_, which remained unchanged in diabetic rats when compared with their age-matched controls. Rupp *et al*. [16] reported that the decrease in myosin ATPase activity may be prevented by treating the CPT-1 inhibitor. Hence, the isoenzyme shift by OXF toward fast myosin V_1_ might be expected to raise *Q*_max_, and thus, decrease LV internal resistance. However, *Q*_max_ did not increase, but decreased with OXF administration to the DM. This result could be explained by the finding that arterial load is also an important factor that inversely affects *Q*_max_ [25], [46]. Our previous study showed that treating with OXF enhanced the already high plasma levels of FFA, which increased *R_p_* in the diabetic peripheral circulation [17]. This elevated *R_p_* may prevail over the isoenzyme shift toward fast V_1_, resulting in a decline in *Q*_max_ (Figure 4B). The diminished *Q*_max_ may augment LV internal resistance and impair the ventricular outflow in insulin-deficient rats. Thus, OXF could not optimize the integrative nature of the cardiac pumping mechanics because the reduced *Q*_max_ counteracted the enhanced *E*_max_ in the DM. By contrast, ALC supplementations did not modify *Q*_max_ in the diabetic animals (Figure 3B). Thus, the enhanced *E*_max_ and unaltered *Q*_max_ from ALC treatment may maintain the optimality of energy transferred from the LV to the arterial system, which is essential for the metabolic needs of tissues and/or organs in diabetes.

As for the diastolic properties of the LV, ALC treatment of the diabetic rats improved LV relaxation in terms of *dP*/*dt*_min_ and τ (Table 3). The similar finding of a significant improvement of cardiac diastolic function has been observed in STZ-diabetic rats administered l-carnitine [47]. Although etomoxir (another CPT-1 inhibitor) was reported to have a selective influence on the rate of relaxation of pressure-overloaded rat heart [48], OXF treatment in this study produced no beneficial effects on either *dP*/*dt*_min_ or τ in the DM.

Based on the findings in this study, there is a good possibility to use ALC to treat patients with metabolic disturbances in diabetic cardiomyopathy. That is because ALC therapy may target those metabolic aberrations in the heart and exert a great benefit to cardiovascular performance. Treatment with ALC significantly reduced abnormalities in lipid profiles, attenuated arterial stiffening and cardiac hypertrophy, and improved myocardial contractility and ventricular relaxation. As for the clinical study with the CPT-1 inhibitor OXF, it was reported to significantly increase the time to onset of angina in patients subjected to progressive pacing stress [49]. However, the drug was shown to damage mitochondrial metabolism, reducing oxygen consumption and uncoupling oxidative phosphorylation in the rat heart [50]. Its development in clinical study was discontinued [51]. Herein, although OXF improved myocardial contractility in diabetes, it exerted biochemical toxicity to the heart, i.e. accumulation of FFA and MDA. Treatment with OXF had no effect on cardiac mass and relaxation function, even deteriorated cardiac output, mean aortic pressure and LV end-systolic pressure. In this informative manner, we presented the clinical differences between the effects of OXF and ALC, suggesting that ALC may be a potential candidate for the treatment of patients with metabolic disturbances.

Certain limitations of this study need to be addressed. Our approach is highly dependent on the elastance-resistance model, which is not a perfect model for the evaluation of LV systolic mechanics. Hunter *et al*. [20] demonstrated that in addition to elastance and resistance, at least 2 or more processes are involved that determine systolic mechanical behavior of the ventricular pump. These processes include the effects of the volume influence factor and the deactivation factor. However, Campbell *et al*. [22] showed that the elastance-resistance model could be used to effectively fit the measured LV pressure of an ejecting beat if the fitting interval is *t_ej_*<*t*<*t_piso_*_max_. Furthermore, Shroff *et al*. [23] believed that the elastance-resistance model is useful for quantifying the systolic pumping mechanics of the LV if one clearly understands its limitations.

Our contribution in this endeavor was to provide a path to consider the clinical application of an elastance-resistance model in the study of cardiodynamics. From the technical point of view, the indispensable isovolumic signals must be obtained by occluding the ascending aorta at the end of diastole, and this measuring technique is not permitted in human subjects. To unravel this serious issue, the isovolumic pressure curve was obtained from the instantaneous pressure of an ejecting contraction by a curve-fitting technique, proposed by Sunagawa *et al.* [28]. Our data showed good quality of the model fit when this elastance-resistance model with the *estimated* isovolumic pressure was applied. The practical advantage of such an approach was that one could compute the ventricular elastance and resistance without any measurements of isovolumic contraction. Moreover, these two cardiac systolic parameters could be calculated from the pulsatile LV pressure and ascending aortic flow signals obtained over a single cardiac cycle without any perturbations of the loading conditions.

Overall, alterations that occurred in the LV included a decline in *E*_max_ in the absence of any significant changes in *Q*_max_ in the STZ-induced diabetic rats. An increase in *V_eed_* might act in concert with the decreased *P_iso_*_max_ and reduce *E*_max_ so that the contractile status of the diabetic heart was impaired. ALC (but not OXF) had reduced plasma FFA levels and cardiac MDA contents in diabetes. However, treating with either ALC or OXF resulted in *E*_max_ increases, which suggests that these 2 drugs may protect the myocardial contractility from deteriorating in rats with insulin deficiency. By contrast, *Q*_max_ decreased with OXF, but not with ALC, augmenting LV internal resistance in diabetes. Moreover, treating the diabetic rats with ALC, but not with OXF, produced a benefit on LV relaxation in terms of *dP*/*dt*_min_ and τ. Thus, its ability to prevent the cardiovascular dysfunction of the diabetic rats allowed ALC, but not OXF, to maintain the optimality of energy transferred from the LV to the arterial system.
